# Ensemble Approach to Combining Episode Prediction Models Using Sequential Circadian Rhythm Sensor Data from Mental Health Patients

**DOI:** 10.3390/s23208544

**Published:** 2023-10-18

**Authors:** Taek Lee, Heon-Jeong Lee, Jung-Been Lee, Jeong-Dong Kim

**Affiliations:** 1 Division of Computer Science and Engineering, College of Software and Convergence, Sun Moon University, Asan 31460, Republic of Korea; jungbini@sunmoon.ac.kr (J.-B.L.); kjd4u@sunmoon.ac.kr (J.-D.K.); 2 Department of Psychiatry, Korea University College of Medicine, Seoul 02841, Republic of Korea; leehjeong@korea.ac.kr

**Keywords:** episode prediction, hidden Markov model, recurrent neural network, random forest, mood disorder, digital healthcare, wearable device, digital phenotype

## Abstract

Managing mood disorders poses challenges in counseling and drug treatment, owing to limitations. Counseling is the most effective during hospital visits, and the side effects of drugs can be burdensome. Patient empowerment is crucial for understanding and managing these triggers. The daily monitoring of mental health and the utilization of episode prediction tools can enable self-management and provide doctors with insights into worsening lifestyle patterns. In this study, we test and validate whether the prediction of future depressive episodes in individuals with depression can be achieved by using lifelog sequence data collected from digital device sensors. Diverse models such as random forest, hidden Markov model, and recurrent neural network were used to analyze the time-series data and make predictions about the occurrence of depressive episodes in the near future. The models were then combined into a hybrid model. The prediction accuracy of the hybrid model was 0.78; especially in the prediction of rare episode events, the F1-score performance was approximately 1.88 times higher than that of the dummy model. We explored factors such as data sequence size, train-to-test data ratio, and class-labeling time slots that can affect the model performance to determine the combinations of parameters that optimize the model performance. Our findings are especially valuable because they are experimental results derived from large-scale participant data analyzed over a long period of time.

## 1. Introduction

There are limitations to managing patients with mood disorders through counseling and drug treatments. Counseling is only meaningful when visiting a hospital, and drug side effects are a burden. Therefore, it is important for patients to understand and manage the conditions causing these episodes. Ultimately, it is important for patients to monitor their mental health status daily and predict and respond to the possibility of an episode occurring. If an appropriate episode prediction tool can be utilized, patients will be able to self-manage their mental health, and doctors will be able to gain insight into the patents’ lifestyle patterns that have worsened the episode.

For patients with mood disorders, a depressive episode is a period in which overall mental and behavioral changes occur along with a decrease in mood. The term episode means that there is a clear distinction between when symptoms are present and when they are asymptomatic. During a depressive episode, the depressed state lasts for most of the day, and this characteristic is very important in distinguishing normal from pathological conditions. Therefore, predicting an episode in real time using digital phenotypic data obtained from a wearable sensor device can help to quickly grasp the condition of the patient in daily life and manage the mental health of the patient.

In a face-to-face clinical assessment of three-month intervals, clinicians determined the onset of recurrent episodes from the previous assessment by reviewing the patients’ mood self-reports and their experienced symptoms. The clinicians were blinded to both the lifelog data collected from the wearable tracking devices and the results of the prediction algorithm. Throughout this process, all patients recorded the timing and duration of their episodes.

Many studies have analyzed or predicted mood changes and stress levels in patients with mood disorders. Such studies have used smartphone sensor data, social media data, and circadian rhythms to predict mood and used heart rate variability (HRV) to assess and predict mental health. These studies primarily performed data analyses using algorithms related to machine learning, deep learning, and regression analysis.

A previous study [1] predicted changes in depressive moods among 31 college students and used passive sensor data collected from smartphones to predict depressive moods over time. The study reported in [2] proposed an online anomaly detection method for the early detection of surgical complications during recovery and the prevention of recurrence in patients with serious mental illnesses. In another study [3], the authors recruited 14 elderly participants living alone and used wearable bands equipped with multiple sensors to monitor their daily activities and biometric data for 71 days. In the study reported in [4], the authors detected changes in depression severity without clinical input data by analyzing accelerometer data from 100 participants. They used a machine learning model to predict clinically relevant changes in depression based on clinical and typing measures. Another previous study [5] collected and analyzed data such as the number of phone calls made to others through smartphones, the number of text messages, and the entropy of changes in the subject’s location based on a global positioning system (GPS). Some studies [6,7] have analyzed human behavioral movements using accelerometer sensors, ambient lighting and noise [6], and paralinguistic features of smartphone voices [7]. In one study [8], a smartphone sensor was used to predict mood changes in 32 subjects over a two-month period. The authors analyzed data on the number and length of phone calls, text messages, and email communications. A mood prediction model was built using app usage patterns, web browser connection history, and location change information; the prediction accuracy of the model was 66%. In the study reported in [6], the mood states of 15 subjects were predicted with a 50% accuracy by analyzing location information, user behavior, ambient light, and sound for 30 days. In another study [9], a project called MONARCA was developed. In that study, 12 patients with bipolar disorder were observed and analyzed over 12 weeks. Using accelerometer sensors and GPS-based location information, a model with a mood prediction accuracy of 72–81% was developed. 

Circadian rhythm mechanisms have been reported to be important factors affecting the onset and exacerbation of mood disorders [10,11,12]. In particular, circadian rhythm misalignment may be a unique clinical sign observed in patients with mood disorders, and studies have shown that changes in the phase of circadian rhythm may be an indicator of mood disorders [13,14]. Circadian disturbances, including diurnal mood variations, have been reported in patients with major depressive disorders, including diurnal mood variation [15]. Another study [16] reported that seasonal variations in mood, behavior, diurnal preference, and irregular bed-rise times are closely related to patients with bipolar disorder.

Sensor data have also been used for health analyses and evaluations in various fields. For example, the study reported in [17] investigated sleep apnea by measuring fluctuations in the average cardiac electrical axis accompanying breathing and respiratory signals derived from the ECG of the body surface. HRV has been suggested to be an important indicator of the mechanistic relationship between mental stress and cardiovascular disease [18].

Various machine learning and deep learning algorithms have been used to predict the health status in various medical fields [19,20,21,22,23,24,25,26,27,28,29]. For example, in the study reported in [24], although it is not directly related to the field of mental health assessment, the authors proposed a method of encoding syntactic knowledge based on long short-term memory for emotion classification. Another study [25] monitored sleep patterns using a bed pressure mat sensor to relieve insomnia. In that study, a pattern recognizer was designed using k-nearest neighbors, the hidden Markov model (HMM), and support vector machine algorithms. In one study [26], people with insomnia were monitored by analyzing radio signals reflected from the body using a device called EZ-sleep. The sleep latency and total sleep time were precisely predicted using the HMM and convolutional neural network (CNN) algorithms. In some previous studies [27,28], linear regression techniques were used to characterize abnormal content in Internet posts to identify pro-eating disorder (ED) content, and a study was conducted to measure the severity of ED for each individual in the pro-ED community. In another study [29], a deep learning model was proposed to identify pro-ED-related articles via automatic pattern modeling of various heterogeneous data such as images and texts posted on social media.

Most existing studies related to mood disorders are small-scale experiments conducted at a laboratory level for limited periods. In contrast, this study is based on a cohort study [30] in which 224 patients were followed up for 1590 days; however, only appropriate data were used. This study accumulated high-quality data from real-life environments, and based on these data, predictive modeling and model performance analysis are different from those of previous studies. The circadian rhythm data are large-scale time-series data captured from sensors. In this study, a prediction model was constructed using the HMM and recurrent neural network (RNN) suitable for modeling time-dependent sequential data. Finally, although the random forest model is not suitable for modeling time-series data, which was proven to some extent in episodic prediction performance in our previous study [30], it was additionally adopted to finally propose an ensemble-type hybrid model to improve performance. 

The contributions of this study are summarized as follows. First, an episode prediction model with satisfactory performance is built based on a long period of actual patient data, and factors affecting model performance are analyzed. Second, features that capture behavioral patterns from sensor sequence data are proposed to help predict episodes. Third, owing to the lack of research on predicting episodic events that will occur in the near future using past time-series behavioral data, algorithms such as the HMM and RNN, specialized for time-series data, are explored to model sequence data from wearable sensors, and their utility is proven through experiments. Finally, an ensemble hybrid model is proposed to increase the model performance.

This study aimed to answer two main research questions: (1) Is it possible to predict whether there will be an episode event in the near future (e.g., the next 3 days) of depressed patients using lifelog time-series data collected from digital device sensors? (2) What is the influence of the factors that affect the performance of the predictive model? Examples of such factors include data-sequence size, ratio of training and test period, and labeling method according to whether an episode event is observed for the following days. The answers to the two questions will be presented in Section 3. 

To verify the hypothesis and answer the research questions, we needed to develop prediction models, and test and compare their performances with a baseline model (hereafter, it will be referred to as the dummy model). Thus, we adopted available diverse models such as HMM [31], RNN [32], and random forest (RF) [30]. HMM offers advantages in predicting depressive episodes by capturing temporal dependencies, handling incomplete or noisy data, and providing interpretable results. Its ability to model sequential data and incorporate uncertainty makes it a versatile and robust tool for analyzing mood patterns and predicting future episodes in individuals with mood disorders. Another promising machine learning tool is RNN. Theoretically, the RNN model is simple and has the advantage that it can handle sequential data of any length. Finally, we also adopted the RF model. In general, although RF is an algorithm that is not suitable for modeling time-series data, it is robust against overfitting, missing values, and outliers. It does not require a normalization process and shows good accuracy even with nonlinear data. Furthermore, it has the advantage of being easy to apply to small-sized data compared to HMM or RNN. Finally, a hybrid model developed by integrating the three models (RF, HMM, and RNN) is presented. The hybrid model worked in an ensemble approach, voted on the prediction results from other models, and was able to improve the performance of other models while supplementing their weaknesses.

## 2. Materials and Methods

This section describes the data collection method and overall experimental design used to test the research hypotheses. The study was approved by the Institutional Review Boards of all the participating hospitals and adhered to the principles outlined in the Declaration of Helsinki. Prior to enrollment, all participants were informed about the study and provided written informed consent.

**Figure 1:**
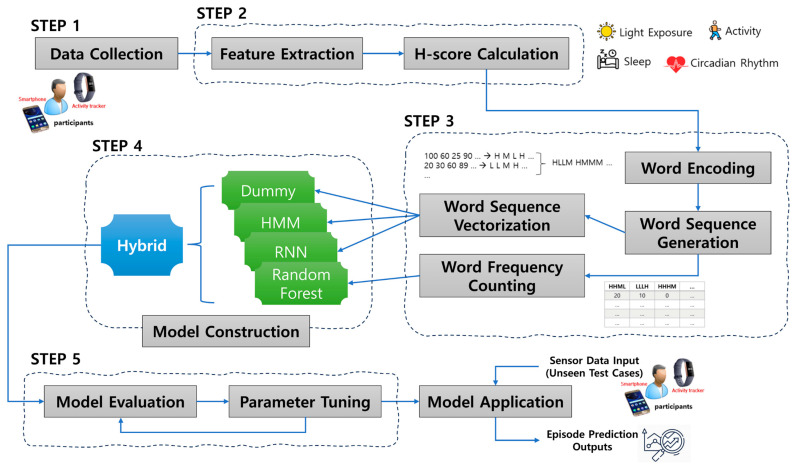
Overview of the experimental process.

In the experiment, we constructed several prediction models and compared their performance with that of the dummy model. In addition, we investigated the factors that affect model performance and conducted experiments to determine the effect of those factors on performance. Figure 1 shows the overall experimental process carried out in this study.

### 2.1. Data Collection (Step 1)

In this study, the adequacy of light exposure, sleep, activity, and circadian rhythm of study participants—the major depressive disorders in patients—were evaluated as daily quantified scores using a smartphone and an activity tracker (Fitbit) to analyze their correlation with the occurrence of future episodes. With the written consent of the participants, data were collected from 224 people over 1590 days; each participant had a different period of participation. The occurrence and timing of depressive episodes were obtained through periodic consultations with the patients by professional medical staff.

### 2.2. Calculation of Health Condition Scores (Step 2)

Light exposure (*f*_1_) can be measured using the smartphone illuminance sensor. Patients receive a higher score (*s*_1_) when as much light as possible is detected in the morning and during the day, as well as the lower degree of light received at night. Sleep (*f*_2_) was measured through an activity tracker worn on the wrist; the higher the sleep efficiency and when the patient is well slept, the higher the score (*s_2_*). Sleep efficiency was calculated through the rate of tossing and turning during the total sleep time, and the sufficiency of sleep was measured by calculating the gap between the recommended sleep of 7 h and 30 min in adults and the total sleep time [33]. The amount of activity (*f*_3_) was also measured through an activity tracker. The more the participant walked during the day and the less the participant walked during the night, the higher the score (*s*_3_). For the circadian rhythm (*f*_4_), the pulse was sampled from an activity tracker for the previous 48 h to infer a daily rhythm of the cosine curve via a cosinor analysis [34], and then a score was given by measuring the parameter acrophase shift from the average. The further from the average (approximately 13–15 h), the lower the score (*s*_4_). For example, in a person who leads an extremely out-of-rhythm lifestyle, such as napping and nocturnal activity, the acrophase is severely misaligned. A more detailed explanation of the four features (*f*_1_–*f*_4_) described above can be found in our prior study [30].

### 2.3. Data Transformation for Machine Learning (Step 3)

HMM and RNN models take sequential data as input. Therefore, the H-score data calculated in STEP 2 above must be observed in chronological order and converted into sequence data. 

For HMM modeling, two HMMs were constructed as the information about the duration of an episode serves as the ground truth. First, pHMM is a positive model built with the feature sequence observed during two weeks before the time *t* of the episodic diagnosis. For a given time point *t*, if an episode period is included between [*t* + 1, *t* + 3], the observation sequence *O* = [*o*_1_, *o*_2_, ..., *o*_14_] is collected for a period [*t* − 14, *t* − 1]. Second, nHMM is a negative model created with feature sequence data for the prior two weeks based on normal days without episodic diagnosis. Thus, given a point in time t, if no episode period is included between [*t* + 1, *t* + 3], the observation sequence *O* = [*o*_1_, *o*_2_, ..., *o*_14_] is collected. Here, two weeks was arbitrarily set because when psychiatrists diagnose depression, they usually observe the condition of the patient for the two weeks before the observation day. In addition, a margin of three days was set to check the duration of the episode. Many cases of the observation sequence *O* for constructing pHMM and nHMM were obtained repeatedly while sliding *t* for the entire data collection period. The element *o* constituting the observation sequence had 81 cases: 4 factors had 3 levels. The factors that determine the element *o* were four scores (s_1_ ~ s_4_) obtained from the four observable features (light/sleep/activity/circadian rhythm mentioned in Step 2), and each score had three levels (high/medium/low). To encode the state of observable features at a given time *t*, words were created using H/M/L alphabetic symbols for each of the feature combination f_1_, f_2_, f_3_, and f_4_. We coined words that express the number of 81 cases like these, for example, LLML, HHHH, HMHM, LLLL, …, and so on. They represented the combination of features observed every day with the form of the word of the day. However, each word was identified with a given unique number in the process of HMM learning. 

For RNN modeling, the word sequences that were input to the HMM obtained above were used as they were. However, since the sequence data input to the neural network needs vectorization, it underwent a word embedding process. If the sequence sizes are different, additional padding is required, but in our experiment, the input data were designed to have the same sequence size, thus padding was not required.

HMM and RNN take sequence data as input, whereas the RF model takes snapshot features at a specific moment as input. Therefore, it is necessary to extract static feature information from given sequence data. For example, for *O* = [*o*_1_*, o*_2_*, ..., o*_14_], there are 81 observable word types (vocabulary) in set *O*. The word frequency can be investigated by counting how many times 81 words individually appear in set O. In this way, a word frequency table can be created by examining the frequency of occurrence of all word vocabularies for given sequences, and an RF can be trained using the table. For class label information, supervised learning is performed using the class information created in the process of generating sequence data for HMM.

When the sequence size was fixed at 14 days, the total sample size of the sequence data for model learning created in Step 3 was 6436, of which 5686 were negative-label samples (euthymic days) and 750 were positive-label samples (episodic days).

### 2.4. Prediction Model Construction (Step 4)

In this step, Python interpreter version 3.9.13, scikit-learn version 1.0.2, hmmlearn library version 0.3.0, and tensorflow version 2.13.0 were used for model construction and experimentation. 

The dummy model was programmed to predict episode events (positive or negative) randomly regardless of the input data. 

HMM assumed two hidden states when building the model. How to build the HMMs used in the prediction phase is as follows: given an observation sequence *O* = [*o*_1_, *o*_2_, ..., *o*_14_] as a test case at a certain time point *t*, the probability that the sequence is observed in either the pHMM or the nHMM model is calculated. That is, if *Prob*(pHMM(*O*)) > *Prob*(pHMM(*O*)), it means that an episode is likely to occur within three days from the date *t*, and in the opposite case, the next three days are likely to be euthymic days.

When building the RNN model, in tuning parameters with experimental experiences, the number of dimensions (embedding_dim) of the word embedding vector used in the tensorflow.keras library was 16, the number of nodes (hidden_units) of one hidden layer was 8, the batch size for training was 32, and the learning epochs were 4. The RF model was built using the default parameter settings provided by the scikit-learn library. The classification probability threshold used in both RNN and RF was set to 0.2.

### 2.5. Model Evaluation and Optimization (Step 5)

To evaluate and compare models, metrics of precision, recall, and F1-score were used because they are popularly used in the machine learning literature [35]. Precision is the number of instances correctly classified as positive, divided by the total number of all instances classified as positive. Recall is the number of instances correctly classified as positive, divided by the total number of actual positive instances. F1-score is a harmonic mean of precision and recall for positive instances [36]. 

To evaluate the performance of the models, several instances were tested by varying the test time point *t*. After splitting the entire data 50:50 over the timeline, the first half of the data were used for learning and the other half were used for testing. Under that setting, the model trained with past data predicts an episode event by looking at an observation sequence at an unseen future time point. 

However, the 50:50 data split between learning and testing based on the time axis is not necessarily fixed; it may be changed. Factors that affect performance must be considered when evaluating models. For example, in the description of Step 3, the sequence data size was assumed to be 14, but this is also a variable. As mentioned above, the split criterion for learning and testing is also a variable factor that affects performance, and the timeslot for the next three days, which is the interval for determining whether future episode events are observed or not, mentioned in Step 4, is also a variable factor with the possibility of change. As mentioned in the second research question in Section 1, the experiment results are introduced in Section 3, and the influence of these variable factors on the model performance is evaluated.

### 2.6. Model Application (Step 6)

In model application, as in model learning, sensor data undergo Steps 2–3, and they are created as a test case of instance data; these are data that have never been seen (unlearned) from the perspective of the model, and when fed into the model, the model estimates and returns the classification probability. The built model uses sequential sensor data from the past 14 days to predict the likelihood of an episodic event occurring in the next 3 days. Here, the sequence data size and the timeslot size for the episode event check are the optimal values obtained through the experiment in Section 3. The model developed from the proposed modeling process (Figure 1) may allow just-in-time event prediction and adaptive interventions to treat depression patients.

## 3. Experiment Results

**Figure 2:**
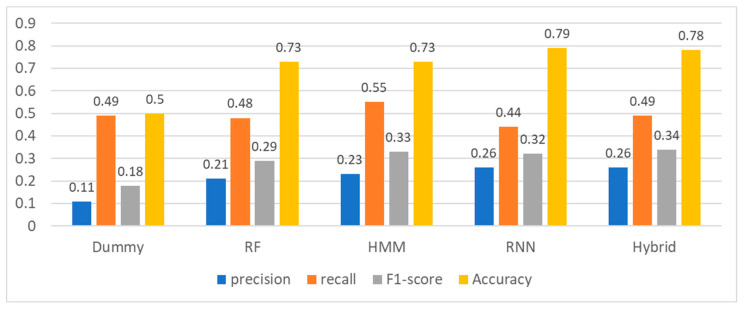
Model performance comparison.

Figure 2 shows the model performance evaluation results. Each of the performance measures is about predicting the positive class label; predicting rare episode events is usually more difficult than common euthymic events. The prediction accuracies of HMM and RNN were 73% and 79%, respectively, significantly outperforming the dummy model and confirming that the first research question is true; the models can predict episode events for the upcoming 3 days using the past 14 days of sequential sensor data. Thus, HMM and RNN can capture chronological patterns in the time-series sensor data, supporting the interesting hypothesis that the current state of health condition can be dependent on the past state of user behavior or habit to some extent.

The prediction accuracy of the hybrid model was 78%, and the F1-score performance was about 1.88 times higher than that of the dummy model (34% vs. 18%). The hybrid model raised the F1-score performance by integrating the other models. Table 1 shows the details of the performance measurement of the hybrid model. For the negative test cases, the model performed quite high (F1-score: 86%), but for the positive test cases, it showed a relatively low performance (F1-score: 34%). This is a phenomenon that occurs because the positive sample ratio (about 11% = 750/6436) in the data pool is relatively low. From a practical point of view, the episode prediction model is as much useful as it predicts positive cases better than negative cases.

Table 2 presents the results of comparing the performance of each model in terms of the F1-score and accuracy. The numbers in the table are multiples of the performance values of each model, assuming that the performance of the dummy model is 1. Table 2 summarizes the advantages and disadvantages of each model.

**Figure 3:**
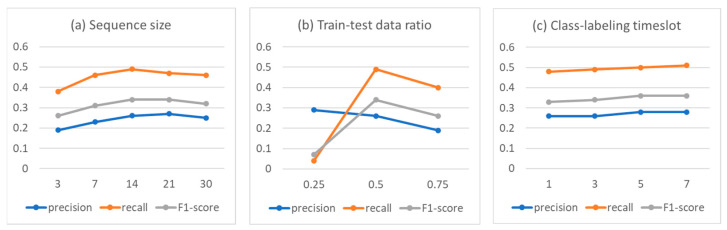
Model performance sensitivity by changing variables: (**a**) sequence size, (**b**) train/test data ratio, and (**c**) class-labeling timeslot.

As discussed in Step 5, several factors affect the performance of the prediction model; we selected three of them. We focus on the data-sequence size, the composition ratio of training and test data, and the future timeslot to determine the positive class label (to check if an episode event exists in the future). Figure 3 shows the results of analyzing the performance sensitivity when the corresponding variable factor was varied while the other two remained constant. In Figure 3, the sensitivity was evaluated based on the hybrid model.

Sequence size (See Figure 3a) was analyzed with the train/test data ratio fixed to 50:50 and the class-labeling timeslot fixed to 3. As the sequence size increased (horizontal axis), the model performance increased at the beginning, peaked at about 14, and then decreased. This implies that it is most efficient to predict the near future by referring to past data of about two weeks. This is consistent with the usual practice by clinicians. In the case of sequence size, it was determined that it was illogical to consider past data of more than one month to realistically predict the near future health status of patients; thus, the change in size was limited to 3 to 30 days.

As seen in Figure 3b, it seems appropriate to maintain the training/test data ratio at 50:50. Even if there is too little or too much training data, it is judged to be not good in terms of performance. In other words, if there is too little data for training and too much data for testing, it will be difficult to predict the future with a poorly trained model. Also, if there is too much training data, the model overfits the data for a long time, and the prediction performance for unseen test cases in the future becomes poor.

In the case of the timeslot that determines the class label (See Figure 3c), since the model predicts the health status of the patient in the near future, the model performance was observed from 1 to 7 days. It was confirmed that the performance increased slightly as the size of the timeslot increased. The reason for this result is that the longer the timeslot, the easier the challenge for the predictive model. This is because it is easier for a model to predict that an episodic event will occur in at least a week than it is to predict that it will occur within a few days. Therefore, in this study, the experiments reported in Figure 2 and Table 1 were conducted under a three-day timeslot.

## 4. Suggestions for Future Research

For patients with mental illnesses, it is difficult to recognize their serious health conditions unless they visit a hospital and consult a doctor. In most cases, when an episode occurs, it is identified later through interview and expert consultation, and action is taken at that later moment. However, it is difficult for medical staff to monitor patients’ health conditions in a timely manner, because they cannot observe patients’ daily life patterns outside the hospital.

Therefore, the episode prediction model proposed in this study monitors patients 24/7 through wearable sensor devices on behalf of medical staff outside the hospital, recognizes patterns of changes in circadian rhythm features, and predicts episode events through a prediction model. It serves as an observation tool suitable for detecting and responding to problems in advance or immediately.

The episode prediction model can be developed into a decision support or an expert system service. In other words, if the accuracy of the prediction is sufficiently high, it will be possible to evolve the model into a helper tool for deciphering mental illnesses, along with the experience of the medical staff. In addition, rather than simply focusing on the results of the prediction (i.e., positive or negative in terms of event occurrence), if the tool can pinpoint the characteristic elements and patterns in the sequence data that produce such results, medical staff can analyze those parts closely to determine in detail why such a phenomenon occurs by conducting additional research. If several years of clinical data are collected from multiple institutions, a more reliable expert system can be implemented.

The episode prediction model can be developed into a decision support tool or expert system; however, the episode prediction model must first be implemented for self-health management purposes rather than for diagnosis. For example, it would be beneficial if users can easily check the possibility of an episode occurring by simply wearing a wearable sensor device and using a smartphone, along with receiving feedback about their current status and what actions to take through an app. Thus, it would be of great help in managing health if people suffering from mental illnesses can visually monitor and improve their mental health through a smartphone app.

For realizing the services described above, several future research topics must be addressed in advance. 

First, it is necessary to improve the performance of episode prediction models. Here, performance means that the model must output accurate prediction results when unknown test case data are input; it also means increasing the robustness of the model so that errors or malfunctions do not occur, even in test cases containing noise or missing values. The accuracy and robustness of the model need to be tested from various demographic perspectives (e.g., gender, age, and occupation). To increase the reliability of the results, the model must be tested using additional data. 

Second, there is a need to further expand the word vocabulary size in the modeling. We adopted a simple approach in terms of word definition; however, we believe that a more detailed vocabulary will be useful for describing longer data sequences. We plan to conduct further research on this topic.

Third, there are fundamental limitations in predicting the likelihood of future episodes using only circadian rhythm signals obtained through sensor devices. For example, genetic and biological factors, medical history, and other environmental factors may play important roles. Therefore, further research is required to design an ensemble model by incorporating possible variables into the prediction model, along with circadian rhythm data, through wearable sensor devices. 

Fourth, it would be helpful to perform cross-validation to prevent overfitting of the model and ensure a generalized performance. The generalized performance of the model could be optimized by learning data from a specific institution and testing patients from other institutions. For this purpose, a more diverse and extensive data collection process is required.

## 5. Conclusions

In this study, we answered the research question that the observation of past lifelog sequence data is effective in predicting depressive episodes in the near future. By proposing the modeling process, the developed hybrid model predicted the future episode events with reasonable performance (78% accuracy). Furthermore, we explored the factors that can affect the model performance (Figure 3). 

Through this study, we found that episodic events in the near future could be predicted using sequential circadian rhythm sensor data. In particular, this was an experimental result derived based on large-scale participant data analyzed over a long period of time, and thus, we believe this work was meaningful and valuable as an empirical study in the relevant literature.

The hypothesis that passive digital phenotype data collected from sensors can be used as a good source for predicting the mental health of patients needs to be continuously investigated. In the future, we intend to conduct additional robust hypothesis verification by implementing more available modeling techniques. In addition, we plan to conduct a hypothesis analysis by grouping patients by different diagnosis types of major depressive and bipolar disorders.

## Figures and Tables

**Table 1:** Performance measurement of the hybrid model.

Class	Precision	Recall	F1-Score	Support
negative	0.92	0.81	0.86	5686
positive	0.26	0.49	0.34	750
accuracy			0.78	6436
macro avg.	0.59	0.65	0.6	6436
weighted avg.	0.85	0.78	0.8	6436

**Table 2:** Performance comparison and pros and cons of each model. The numbers are multiples of the performance value, assuming that the performance of the dummy model is 1.

	F1-Score Multiple	Accuracy Multiple	Pros	Cons
RF	1.611	1.46	Applicable to small data. Decent performance. Model learning is fast.	Sequence data modeling is not possible
HMM	1.833	1.46	Sequence data can be learned. Small data can be applied.	Difficulty in modeling long sequence patterns
RNN	1.778	1.58	Specialized in sequence data learning.	Training requires a lot of data and a long time
Hybrid	1.889	1.56	Overcomes performance shortcomings of other models. Typically high generalized performance.	Multiple learning models are needed. Does not always guarantee higher performance than individual models

## Data Availability

Data are unavailable due to privacy or ethical restrictions.

## References

[1] Jacobson N.C., Chung Y.J. (2020). Passive sensing of prediction of moment-to-moment depressed mood among undergraduates with clinical levels of depression sample using smartphones. Sensors.

[2] Liu G., Onnela J.P. (2022). Online anomaly detection for smartphone-based multivariate behavioral time series data. Sensors.

[3] Choi J., Lee S., Kim S.Y., Kim D.I., Kim H.S. (2022). Depressed mood prediction of elderly people with a wearable band. Sensors.

[4] Ross M.K., Tulabandhula T., Bennett C.C., Baek E.G., Kim D.H., Hussain F., Demos A.P., Ning E., Langenecker S.A., Ajilore O. (2023). A novel approach to clustering accelerometer data for application in passive predictions of changes in depression severity. Sensors.

[5] Madan A., Cebrian M., Lazer D., Pentland A. (2010). Social sensing for epidemiological behavior change. Proceedings of the 12th ACM International Conference on Ubiquitous Computing.

[6] Ma Y., Xu B., Bai Y., Sun G., Zhu R. Daily Mood Assessment Based on Mobile Phone Sensing. Proceedings of the 2012 Ninth International Conference on Wearable and Implantable Body Sensor Networks.

[7] Calvo R., D’Mello S. (2010). Affect detection: An interdisciplinary review of models, methods, and their applications. IEEE Trans. Affect. Comput..

[8] LiKamWa R., Liu Y., Lane N.D., Zhong L. (2013). MoodScope: Building a mood sensor from smartphone usage patterns. Proceedings of the 11th Annual International Conference on Mobile Systems, Applications, and Services.

[9] Gravenhorst F., Muaremi A., Bardram J., Grünerbl A., Mayora O., Wurzer G. (2014). Mobile phones as medical devices in mental disorder treatment: An overview. Pers. Ubiquitous Comput..

[10] Malhi G.S., Kuiper S. (2013). Chronobiology of mood disorders. Acta Psychiatr. Scand..

[11] McClung C.A. (2007). Circadian genes, rhythms and the biology of mood disorders. Pharmacol. Ther..

[12] Cho C., Lee H. (2018). Why do mania and suicide occur most often in the spring?. Psychiatry Investig..

[13] Moon J., Cho C., Son G.H., Geum D., Chung S., Kim H. (2016). Advanced circadian phase in mania and delayed circadian phase in mixed mania and depression returned to normal after treatment of bipolar disorder. EBioMedicine.

[14] Cho C., Moon J., Yoon H., Kang S., Geum D., Son G. (2016). Molecular circadian rhythm shift due to bright light exposure before bedtime is related to subthreshold bipolarity. Sci. Rep..

[15] Murray G. (2007). Diurnal mood variation in depression: A signal of disturbed circadian function?. J. Affect. Disord..

[16] Geoffroy P.A., Bellivier F., Scott J., Etain B. (2014). Seasonality and bipolar disorder: A systematic review, from admission rates to seasonality of symptoms. J. Affect. Disord..

[17] Moody G.B., Mark R.G., Bump M.A., Weinstein J.S., Berman A.D., Mietus J.E. (1986). Clinical validation of the ECG-derived respiration (EDR) technique. Comput. Cardiol..

[18] Akselrod S., Gordon D., Ubel F.A., Shannon D.C., Berger A.C., Cohen R.J. (1981). Power spectrum analysis of heart rate fluctuation: A quantitative probe of beat-to-beat cardiovascular control. Science.

[19] Sükei E., Norbury A., Perez-Rodriguez M.M., Olmos P.M., Artés A. (2021). Predicting emotional states using behavioral markers derived from passively sensed data: Data-driven machine learning approach. JMIR mHealth uHealth.

[20] Ip E.H., Zhang Q., Schwartz R., Tooze J., Leng X., Han H., Williamson D.A. (2013). Multi-profile hidden Markov model for mood, dietary intake, and physical activity in an intervention study of childhood obesity. Stat. Med..

[21] Garavand A., Behmanesh A., Aslani N., Sadeghsalehi H., Ghaderzadeh M. (2023). Towards Diagnostic Aided Systems in Coronary Artery Disease Detection: A Comprehensive Multiview Survey of the State of the Art. Int. J. Intell. Syst..

[22] Hosseini A., Eshraghi M.A., Taami T., Sadeghsalehi H., Hoseinzadeh Z., Ghaderzadeh M., Rafiee M. (2023). A mobile application based on efficient lightweight CNN model for classification of B-ALL cancer from non-cancerous cells: A design and implementation study. Inform. Med. Unlocked.

[23] Gheisari M., Ebrahimzadeh F., Rahimi M., Moazzamigodarzi M., Liu Y., Pramanik P.K.D., Heravi M.A., Mehbodniya A., Ghaderzadeh M., Feylizadeh M.R. (2023). Deep learning: Applications, architectures, models, tools, and frameworks: A comprehensive survey. CAAI Trans. Intell. Technol..

[24] Huang M., Qian Q., Zhu X. (2017). Encoding syntactic knowledge in neural networks for sentiment classification. ACM Trans. Inf. Syst..

[25] Metsis V., Galatas G., Papangelis A., Kosmopoulos D., Makedon F. (2011). Recognition of sleep patterns using a bed pressure mat. Proceedings of the 4th International Conference on Pervasive Technologies Related to Assistive Environments.

[26] Hsu C.Y., Ahuja A., Yue S., Hristov R., Kabelac Z., Katabi D. (2017). Zero-effort in-home sleep and insomnia monitoring using radio signals. Proc. ACM Interact. Mob. Wearable Ubiquitous Technol..

[27] Chancellor S., Pater J.A., Clear T., Gilbert E., Choudhury M.D. #thyghgapp: Instagram content moderation and lexical variation in pro-eating disorder communities. Proceedings of the 19th ACM Conference on Computer-Supported Cooperative Work & Social Computing.

[28] Chancellor S., Lin Z.J., Choudhury M.D. “This post will just get taken down”: Characterizing removed pro-eating disorder social media content. Proceedings of the 2016 CHI Conference on Human Factors in Computing Systems.

[29] Chancellor S., Kalantidis Y., Pater J.A., Choudhury M.D., Shamma D.A. Multi-modal classification of moderated online pro-eating disorder content. Proceedings of the 2017 CHI Conference on Human Factors in Computing Systems.

[30] Lee H.J., Cho C.H., Lee T., Jeong J.G., Yeom J.W., Kim S.J., Jeon S.H., Seo J.Y., Moon E.S., Baek J.H. (2022). Prediction of impending mood episode recurrence using real-time digital phenotypes in major depression and bipolar disorders in South Korea: A prospective nationwide cohort study. Psychol. Med..

[31] Rabiner L.R. (1989). A tutorial on hidden Markov models and selected applications in speech recognition. Proc. IEEE.

[32] Suhara Y., Xu Y., Pentland A.S. DeepMood: Forecasting Depressed Mood Based on Self-Reported Histories via Recurrent Neural Networks. Proceedings of the 26th International World Wide Web Conference.

[33] Hirshkowitz M., Whiton K., Albert S.M., Alessi C., Bruni O., DonCarlos L., Hazen N., Herman J., Hillard P.J.A., Katz E.S. (2015). National Sleep Foundation’s updated sleep duration recommendations: Final report. Sleep Health.

[34] Moškon M. (2020). CosinorPy: A python package for cosinor based rhythmometry. BMC Bioinform..

[35] Alpaydin E. (2010). Introduction to Machine Learning.

[36] F1-Score (Wikipedia). https://en.wikipedia.org/wiki/F-score.

